# Exploring perspectives on health professions education scholarship units from sub-Saharan Africa

**DOI:** 10.1007/s40037-020-00619-8

**Published:** 2020-09-15

**Authors:** Susan van Schalkwyk, Bridget C. O’Brien, Cees van der Vleuten, Tim J. Wilkinson, Ilse Meyer, Anna M. S. Schmutz, Lara Varpio

**Affiliations:** 1 Centre for Health Professions Education, Faculty of Medicine and Health Sciences, Stellenbosch University, Stellenbosch, South Africa; 2 Center for Faculty Educators, University of California, San Francisco, School of Medicine, San Francisco, CA USA; 3 Department of Educational Development and Research, Maastricht University, Maastricht, The Netherlands; 4 University of Otago Medical School, Otago, New Zealand; 5 Department of Medicine and Center for Health Professions Education, F. Edward Hébert School of Medicine, Uniformed Services University of the Health Sciences, Bethesda, MD USA

**Keywords:** Health professions education, Scholarship, Sub-Saharan Africa

## Abstract

**Introduction:**

There has been a marked increase in institutional structures developed to support health professions education scholarship recently. These health professions education scholarship units (HPESUs) engage in a diverse range of activities. Previous work provided insight into factors that influence the functioning of such units, but data from European, Asian, Latin American, and African contexts was absent, potentially leading to a single world-view informing international standards for HPESUs. This aim of this study was to explore perspectives from sub-Saharan Africa (SSA) in response to this omission.

**Methods:**

Situated within an interpretivist paradigm, the research team conducted semi-structured interviews with nine HPESU leaders in SSA, exploring how participants experienced and understood the functioning of their units. Despite efforts to have representation from across the region, most participants were from South Africa. The researchers analysed data thematically using the theory of institutional logics as an analytical frame.

**Results:**

Several aspects of the HPESUs aligned with the previously identified logics of academic research, service and teaching; and of a cohesive education continuum. By contrast, leaders described financial sustainability as a more prominent logic than financial accountability.

**Discussion:**

The similarities identified in this study may reflect isomorphism—a process which sees institutions within a similar field becoming more alike, particularly as newer institutions seek to acquire legitimacy within that field. An important caveat, however, is that isomorphism tends to occur across similar institutional contexts, which was not the case in this study. Understanding these differences is key as these HPESUs move to foster scholarship that can respond to the region’s unique context.

## Introduction

Health professions education (HPE) has recently witnessed a marked increase in the number of institutional structures developed to support scholarship in the field [1–6]. A growing body of research is investigating health professions education scholarship units (HPESUs), defined as organizational structures actively engaged in HPE scholarship [7]. This research highlights how each HPESU embraces a unique configuration of operational scopes, organizational configurations, and purposeful foci of activities and research [4–7]. An exploratory study of HPESUs in Canada, Australia, New Zealand, and the USA [5] constructed an understanding of how three institutional logics underpinning HPESUs—that is, the norms and values that are shaped over time and come to characterize an organization—influence the way in which these units function. The logics guiding HPESUs in these nations were: financial accountability; a cohesive education continuum; and competing demands of academic research, service and teaching [5]. While this work offers an international perspective on HPESUs, the authors acknowledged the absence of data from European, Asian, Latin American, and African contexts. Without insights from these other settings, the research promotes a single world-view and risks becoming embraced as an international standard for all HPESUs. We aimed to respond to this omission by exploring the institutional logics common across HPESUs in sub-Saharan Africa (SSA).

Following global trends, SSA is increasingly participating in HPE, supported by an expanding number of medical schools, especially since 1990 [8]. In 2010, the newly developed Medical Educational Partnership Initiative (MEPI) grant [9] made US$ 132 million available to support medical education in SSA over a 5-year period (2010–2015). Simultaneously, the Foundation for the Advancement of International Medical Education and Research (FAIMER) and its regional affiliate, the Southern African FAIMER Regional Initiative, have catalyzed the establishment of HPESUs in the region [3]. However, SSA’s HPESUs are faced with challenges unique to the region—e.g., the burden of disease (SSA bears one quarter of the world’s total disease burden) and significant constraints in terms of resources (e.g., SSA has less than 4% of the world’s total healthcare workforce) [10]. The region lags behind much of the world with respect to the provision of healthcare [11–13]. This situation is exacerbated by limited resources and significant workforce shortages, which impacts the provision of quality education and training for healthcare professionals. Given these considerations, it is reasonable to expect that SSA’s HPESUs will differ from those previously studied [4–7].

The purpose of this investigation was to construct an understanding of the ways in which HPESUs function in SSA and of the perspectives that influence their practices in this region. Two research questions fuelled our investigation:

1. What are the structural and organizational properties of SSA’s HPESUs?
2. What are the institutional logics influencing the development of SSA’s HPESUs?

## Methods

We intentionally adopted a methodology similar to that employed in the earlier studies to allow for comparative reflection. This study was situated within an interpretivist paradigm, applying thematic analysis to identify, analyze, and report patterns of meaning in the qualitative data set [14–16]. Thematic analysis is not a research methodology; instead, it is a method for data analysis that can be tailored to the ontological and epistemological roots of a variety of research paradigms. When used from within the interpretivist orientation, thematic analysis can help researchers gain insight into the social, cultural, and structural contexts that influence individuals’ experiences [16].

### Context

The African continent comprises 58 countries [17]; SSA, made up of 51 countries, refers to that part of the continent that lies fully, or partially, south of the Sahara Desert [17]. The number of medical schools in SSA has increased rapidly in recent years [18], although determining the precise number of medical schools is problematic because comprehensive information across all 50 nations is not readily available, nor is it consistently updated. Nevertheless, a sub-Saharan African medical schools survey, conducted between 2008 and 2009, identified 169 medical schools in the region, with 58 having been established between 1990 and 2009 [10, 19]. At present, information about which SSA medical schools house an HPESU is not available.

### Participants

Since a registry of HPESUs in SSA is not available, we began our investigation by engaging in an HPESU census activity emailing 25 MEPI and FAIMER partners in the SSA region, requesting information on existing HPESUs. Only four responses were received. To support the census, a member of our research team (IM) conducted an internet search of all medical schools listed in the region to learn which had HPESUs. We identified 28 units that, based on the available information, could be categorized as an HPESU. These units were located in 14 different SSA countries: Botswana, Ethiopia, Ghana, Kenya, Malawi, Namibia, Nigeria, Rwanda, South Africa, Sudan, Tanzania, Uganda, Zambia, and Zimbabwe.

The heads of these units were contacted via email, and invited to participate in an online interview. Despite multiple communications, many did not respond; ultimately nine individuals participated in the study. While we cannot claim to have maximized diversity in the sampling process, our pragmatic approach met most of the criteria for information power identified by Malterud et al. [20] by: presenting a focussed study aim; generating high-quality dialogue; using evolving, if not established, theories; and having a clear intent to explore positions through our analysis. All participants provided informed consent, and ethical approval was obtained in March 2018 from Stellenbosch University’s Health Research Ethics Committee (N18/01/008).

### Data collection

One author (IM) conducted all interviews following a semi-structured interview protocol, developed by Varpio et al. [5] and modified by two members of the research team (SvS and LV) to align with SSA contexts. IM has no relationship with any of the interviewees. Interviews occurred from May to July 2018 using an online, audio-only platform, were conducted in English, and lasted 45–60 min.

The interviews were digitally recorded, transcribed, and assigned a code by a third party. Participants’ identifying codes were maintained by IM to facilitate accurate cross-referencing. IM reviewed the transcripts against the original recording to confirm accuracy and ensure anonymity.

### Data analysis

The research team followed Braun and Clarke’s six phases of thematic analysis [14]. First, two members of the research team (IM and AS) independently reviewed the transcripts to familiarize themselves with the data (phase 1). Following an inductive and iterative process, they independently identified relevant features in the data, collating data into codes (phase 2) and then collaborated to search for common themes that ran across the coded data (phase 3). Next, two other members of the team (SvS and LV) reviewed this initial analysis, studying the coded data extracts and the entire data set to confirm the thematic mapping (phase 4). Discrepant interpretations and understandings were discussed until consensus was achieved, including the names and definitions of each theme (phase 5). In phases 4 and 5, the identified themes were considered in light of the theory of institutional logics and the logics common across some nations’ HPESUs previously reported [5]. During the development of this manuscript, all team members reviewed selected data excerpts and interpretations of data to ensure that the analysis robustly addressed the study’s research questions (phase 6).

### Trustworthiness and reflexivity

To ensure transparency and to develop an audit trail of research developments, the research team documented the entire research process, including the development of the potential participants’ database, the interaction with these participants for data collection, the data analyses (including the various iterations of the development of the thematic structure), and the engagement among the different co-authors. Our team represents a wide range of expertise and experience in HPE scholarship. Three authors lead an HPESU (CV, SvS, TW), while the remaining authors are involved in their local HPESUs and participated in some of the earlier studies on HPESUs mentioned previously. Their diverse international perspectives aided the identification of similarities and differences between the HPESUs included in this study and those described in prior work.

However, we grappled with the fact that our research team includes authors from only one SSA unit. We recognize that this impacts the analysis of our data. Further, we acknowledge the limitation of our participant sample in spite of concerted attempts to extend it. By calling attention to the silence of these important voices, both in our research team and in our sample, and acknowledging our discomfort with this, we hope to encourage further discussion of ways to make our future research more inclusive.

## Results

We report our findings by describing the structural and organizational properties of the nine units represented in the data (Tab. 1). Next, we present our thematic analysis by framing our findings in relation to the institutional logics identified as relevant to many international HPESUs [5] but leave comparisons to previous research for the Discussion.

**Table 1:** Characteristics of the health professions education scholarship units

Unit characteristics	No. of units
*Unit names and descriptors*	
Department of Health Sciences Education (fully fledged academic departments within the relevant health sciences or medical faculty)	3
Center for Health Professions Education (autonomous entity within the relevant health sciences or medical faculty which may or may not have academic status)	5
Division Health Sciences Education (situated within a department)	1
*Country*	
Botswana	1
South Africa	6
Uganda	1
Zimbabwe	1
*Unit director’s highest degree*	
PhD	7
MB, ChB (Medical Degree)^a^, Master’s degree in Medicine^b^	2
*Qualifications of unit members*	
PhD = 2; Masters = 4	1
PhD = 3; Masters = 1	1
PhD = 3; Masters = 1	1
PhD = 3	1
PhD = 2	2
PhD = 1	2
Medical Doctor = 1	1
*Unit reports to:*	
Deputy Dean/Vice-Dean/Assistant Dean for education/teaching and learning typically in a medical or health sciences faculty	5
Principal of College/Dean of Faculty (medicine and/or health sciences)	3
Deputy Vice-Chancellor (University leader: teaching and learning)	1
*Units’ funding sources*	
Fully funded by the institution	6
Partially funded by institution, partially funded from other sources	3
*Unit history*	
New director (past 1–2 years) for new or reorganized unit	5
Experienced director (4 years or more) of an established or reorganized unit	4

^a^ MB, ChB (6-year undergraduate qualification in Bachelor of Medicine and Bachelor of Surgery)

^b^ Master’s in Medicine (requirement to become a specialist in SSA, equivalent to a consultant)

### The structural and organizational properties of the HPESUs

Six of the nine study participants worked in HPESUs within South Africa’s national borders, and three were located in Botswana, Uganda, and Zimbabwe. None of the units had been in existence prior to 2000. Tab. 1 details the structural and organizational properties of the nine HPESUs represented by study participants.

### The institutional logics influencing the development of the HPESUs

Participants’ responses addressed the previously reported logics of academic research, service, and teaching; of the educational continuum; and of financial accountability.

#### The logic of academic research, service, and teaching.

The logic of academic research, service, and teaching was clearly evident in participants’ descriptions of their unit’s activities. Tab. 2 provides an overview of each unit’s activities as described by participants. The participants described how their local HPESU engaged in activities related to service, teaching, and academic research; however, one unit had a more limited scope of activities than the others and did not define HPE research as a key unit activity. While there was considerable variability between units, four service activities were consistently highlighted as important components of the HPESU’s mandates. These four activities were: supporting staff/faculty development; supporting e‑learning/educational innovation; supporting teaching staff in education-related endeavors (e.g., curriculum development or renewal); and supporting students’ learning activities at both undergraduate and postgraduate levels.

**Table 2:** Key activities of each health professions education scholarship unit (*HPESU*)

Activity	HPESU								
	1	2	3	4	5	6	7	8	9
Staff/faculty development	x	x	x	x	x	x	x	x	x
Support for e‑learning/educational innovation	x	x	x	x	x	x	x	x	x
Postgraduate programme offerings	x	x	x	–	x	–	–	x	–
Quality assurance	–	–	–	–	–	–	x	x	x
Support for staff in education-related endeavors (i.e., curriculum renewal)	x	x	x	x	x	x	x	x	x
Student support activities	x	x	x	x	x	x	x	x	x
Promote the development of HPE scholarship within their institutions	x	x	x	x	x	–	x	x	x
Engage in their own HPE research	x	x	x	x	x	–	x	x	x

In relation to a service-oriented focus, offering faculty development activities was a common service-related activity described by participants. The extent to which there was engagement in such offerings—i.e., the degree to which “academics buy into the staff development support initiatives” (#4)—was frequently identified as a measure of the unit’s success. Furthermore, despite being relatively small in size (i.e., generally having fewer than 10 members therein), the participants articulated that, via the HPESU’s activities, they aimed to positively influence the standing of learning and teaching in their faculties. The ability to realize this influence was described by participants as connected to the HPESU having “a formal structure” (#7) within the institution, and being valued for the role they fulfilled therein:

*We are appreciated; what we do is taken seriously.* (#9)

*I think our contribution there is that we have got recognition … we are pretty much looking after the staff here*. (#3)

The potential to influence the standing of learning and teaching within the institution was often framed as reliant on either a particular individual or a small cluster of individuals. These individuals were labelled as champions who could act as mentors for staff, and who could garner support for HPE and advocate for learners and teachers:

*Yes, it’s [being] a role model. Some of the people said it’s the smell of the tribe, or the group. So, to follow that smell, and then you will get your champions, you will get people, even in clinicians, that are very interested in medical education, and realize the importance of medical education*. (#8)

Regarding teaching-focused activities, in contexts where the HPESU had a strong academic remit (i.e., where they were responsible for programs leading to a formal academic qualification and/or generally actively involved in academic activities within their institutions), the units were well positioned to influence practice and policy around teaching. Participants described a range of activities that related directly to the institution’s teaching mandate, including serving on key decision-making structures:

*I chair the faculty’s assessment committee … convener of the intervention program … involved in workshops … involved in revision of our ad hominem promotion criteria, by which staff can apply for promotion each year, making sure that teaching and assessment are adequately represented there and scholarship of teaching … run the faculty’s annual teaching conference … consultancy role … accreditation visits … .* (#1)

HPESUs were also involved in different ways in undergraduate (e.g., teaching Philosophy of Health Sciences courses) and postgraduate teaching, including running Masters and PhDs in HPE programmes.

Promoting scholarship and conducting research in HPE were also features of the work of these units. In fact, research was seen as key to garnering status locally, with the number of research outputs highlighted as an important metric of the HPESU’s success. However, engagement in research was not solely focused on disseminating research findings. It was also centered on bringing research evidence back to the institution to enhance local practices: “We present at national, international and local levels, making sure that we also bring back the latest things related to medical education.” (#8).

Managing the broad remit of service, teaching, and research was often challenging; participants expressed concern that the HPESUs could not always drive their own agenda. This led to tension between scholarship—which typically provides tangible outputs against which units could be measured—and service—which often involved more administrative functions for which there were no tangible outputs to be quantified:

*What impedes our success is this diffusion of focus … instead of driving innovation and driving research in health professions education, we are forced to move into a more supportive unit … writing policies and memos*. (#5)

Addressing the tension between service and scholarship was an important aspiration for several of the respondents. All participants spoke appreciatively about future potential, keen to be seen to be “making a difference” (#5) and seeking a shift from focussing solely on service work to adopting a more research-oriented mandate where HPE was recognized institutionally, legitimated as “a field of endeavour” (#1):

*One of the biggest issues that we are working on is exactly the mind-set that was responsible for the success of the units in the past. So that very passion and commitment to the educational development work comes with a service-oriented mind-set and changing that orientation from an exclusively service orientation within the faculty to a more general, if you will, academic orientation [is our aim]*. (#1)

Here it should be noted that, regionally, educational development work has historically been cast as “academic support” with staff in many university centers concentrating on teaching and learning not having academic appointments and, therefore, not necessarily being supported to engage in scholarship. Establishing an HPESU as an academic entity was a key enabler:

*Certainly the commitment of the faculty to establish the [HPESU as] academic department has been there. We have, certainly nominally we have immense support from the leadership in the faculty for the department*. (#1)

*The most significant success in the development of the unit was that it was given academic status*. (#2)

In sum, participants described having to navigate tensions across the logic of service, teaching, and research in their local contexts. While some HPESUs privileged research, this was often accompanied by significant emphasis on metrics and outputs. Individual working in the units worked to strengthen teaching and learning agendas, particularly if they were granted membership on different key institutional committees.

#### The logic of a cohesive education continuum that spans the professions.

Respondents described directing their activities in support of both medical undergraduate and postgraduate levels; however, despite the service activities reported, participants did not explicitly reference engaging in continuing medical education as might be the case in certain contexts. In some instances, the units were heavily involved with undergraduate medical education starting with admissions:

*We consulted widely in the faculty, and our unit drew up the new admissions policy. So, that’s our role in admissions. Undergraduate education, our role is huge. That is where our major emphasis has been*. (#9)

Importantly, participants did not articulate their units as being restricted to supporting only one part of the educational continuum. For example, while participant #9 noted the local HPESU’s heavy involvement in undergraduate medical education, this respondent went on to say:

*In terms of postgraduate education, we are just about to start on a similar exercise … Our involvement with them will increase exponentially*. (#9)

Importantly, in addition to the broad medical education engagement, participants clearly explained that all of the HPESUs also focused their efforts across all professions. Efforts to facilitate this health professions focus included, for example, participating on cross-cutting education-related committees:

*We work right across all the different schools … . We sit on all the committees in faculty, and also at the university, as well as on the committees of the various schools*. (#8)

In fact, participants commonly described engaging in HPE rather than medical education. This focus influenced the approach adopted by the units:

*We probably don’t engage with them all equally, and we don’t have a mandate to engage with some over others. We prefer to talk health science education and not medical education because we see ourselves as being here for the faculty, and not just for the medical program*. (#3)

*Our notion and belief is to have an all-embracing approach, health professions education … . We are not only focussing on medical training, and we emphasize the aspect of IPE, interprofessional education*. (#7)

Therefore, while engagement with the medical education continuum was framed in terms of medical students and residents, the HPESUs’ efforts were not medicine centric. Instead, the HPESUs engaged with many health professions.

### The logic of financial sustainability

Participants noted the importance of finances to the work of the HPESU. Specifically, our data pointed to a focus on financial sustainability. The respondents generally described funding as a necessary condition—but an unstable one—for the HPESUs’ sustainability. The lack of secure funding, particularly the vagaries of external funding, was seen as a significant risk to the HPESU. This had a profound effect on staff, many of whom were on soft funding and short-term contracts, which also limited the potential to attract qualified staff to the unit:

*We are way too few people*. (#5)

*The main impediment for me now is to get the necessary human resources to really have impact*. (#4).

Furthermore, staff working within the HPESU sometimes had dual appointments—one with the HPESU and one with another academic department. This was described as a “messy” (#1) situation that placed multiple demands on staff. Not being able to do “all the work that’s on [our] table” (#5) was a source of frustration for participants. Respondents described a desire to establish collaborations with other institutions, but they lamented not having the resources to do so.

Several units had been either established or strengthened as a result of significant United States government’s funding of the MEPI (see https://www.fic.nih.gov/Programs/Pages/medical-education-africa.aspx) grant:

*When the MEPI grant became available … there suddenly was an opportunity to set up a department. So, what happened was there was a cohort of people who did the SAFRI course, and then the Dean and the Deputy Dean were among those. Essentially they drove the setting up of the department, [but] because we were set up as part of the MEPI, … there was a very big question hanging over us about whether we would be sustainable, post MEPI*. (#6)

Strategies to strengthen sustainability included working directly within academic departments—i.e., to “convert the disciplinary experts into expert teachers of the discipline”—thereby diversifying the disciplinary representation in the HPESU, albeit on an ad hoc basis. While participants spoke about having had an impact on the institution in a variety of ways (e.g., “as an example of how teaching and learning innovation should be driven” (#5)), the nature of the work that the units believed would ensure their sustainability—including via sustained funding—covered the entire spectrum from service to scholarship. For some, financial sustainability could be secured by being “very prolific in our research … known for making sure that our stuff gets published” (#8). Others sought financial sustainability by securing a formally recognized structure within the institution as a unit that would work towards “ensuring total quality improvement” (#7) including “a monitoring and evaluation system” (#7).

## Discussion

An initial catalyst for this study was the assumption that the norms and values that characterize HPESUs in SSA—i.e., the institutional logics—might differ from those previously identified in studies conducted in higher-income countries. However, we found greater alignment between our study data and the findings from published research investigating HPESUs than we expected, specifically in terms of the core activities of the HPESUs. We did, nevertheless, identify some differences. Unit leaders highlighted engaging with the continuum of learners from across multiple professions, not just medicine. Further, the effect of the resource constrained context appears to have directed the logic of financial matters towards sustainability, rather than accountability. Previous studies of HPESUs emphasize how funding sources drive reporting structures, highlighting to whom a unit was accountable [5, 7]. These studies noted that the sources of an HPESU’s funding were not always stable or consistently maintained, creating a situation where the size and mission of an HPESU would grow or shrink as funding changed over the years. The participants in our research highlighted that funding is a foundational lifeline for HPESUs included in this study. Initiatives like MEPI supported the launch of HPESUs [3], but once those funds were depleted, the unit’s continued existence could be threatened. In other words, funding not only affected the size and mission of SSA’s HPESUs, it impacted the ability of the HPESU to survive.

To understand these similarities and differences with other HPESUs—despite the very different regional contexts surrounding them—required us to extend our theoretical net. To enhance our inquiry, we adopted a theory-informing inductive research approach [21] and sought out other theories that could help us explain this phenomenon. This search led us to the concept of isomorphism from the organizational theory that gave rise to the concepts of institutional logics. In the early 1980s, DiMaggio and Powell spoke of the “startling homogeneity” [22, p. 148] that existed among institutions in a particular field (in our case, HPESUs). They drew on the concept of isomorphism, which they defined as a “process that forces one unit in a population to resemble other units that face the same set of environmental conditions” (p. 149). They foregrounded three mechanisms of isomorphism—coercive, mimetic, and normative—that encourage homogeneity. Coercive isomorphism highlights the pressure placed on institutions to conform to acquire legitimacy in the field. Mimetic isomorphism occurs when the context is uncertain and the purpose unclear so institutions copy other institutions perceived to be successful. Normative isomorphism involves the push towards professionalization [22, 23], in this case of the health professions educator. These three mechanisms provide a lens through which we might better understand our study’s findings.

Firstly, coercive isomorphism was evident in the imperative for legitimacy. Institutional logics are intended to offer criteria for legitimacy [5], and legitimacy comes from belonging, meeting societal expectations and standards [22]. What was evident in our data was that legitimacy is still sought by the HPESUs our participants worked within. Participants lamented that their units had yet to be valued by their local faculties and so could be seen as being coerced to adopt the standards and expectations set by the local institution—including standards and expectations associated with academic research, service, and teaching. Secondly, mimetic isomorphism was reflected in participants’ concern about uncertainty of purpose. Several responses implied an evolving rather than an established remit for the units. Others described having to diffuse their focus. While there was no overt evidence of them intentionally seeking to mimic other HPESUs that they deemed successful, it was clear that they had a sense of where they would wish to be that was derived from their understanding of what a successful HPESU would look like. Normative isomorphism was evident in relation to units’ aspirational underpinnings, as respondents described intentionally working towards extending scholarly endeavours. Such focus on growing and strengthening scholarship has been directly linked to the professionalization of HPE globally [24, 25]. In these ways, all three of the mechanisms that influence isomorphism are present in our data.

However, while isomorphism may explain some of the similarities between the logics identified in previous research and those we found in our data, our analysis also highlighted significant differences. These disparities could potentially be ascribed to a caveat that DiMaggio and Powell added to their initial definition of isomorphism, namely that the units seeking to resemble one another are often situated within similar contexts [22]. Such similarity could at best be regarded as tenuous within our study sample, and even more questionable when extended to the HPESUs investigated in other studies. Clearly, additional inquiry is required. Important research directions might shift to: whether, in light of SSA’s limited resources, homogeneity is possible, or even desirable; to what extent HPESUs across the world should function similarly to one another and what dangers might lie in such mimicry? Answers to such questions would help ensure that HPESUs harness best practices from a variety of locations, while simultaneously attending to the unique aspects of their contexts and institutions to understand the logics that underpin each unit’s activities and priorities.

There are other limitations to this study. As noted earlier, and despite our best efforts, we interviewed representatives from less than half of the identified HPESUs in SSA with such representation skewed towards South Africa. While we acknowledge that wider representation would have enriched our findings, and recognize that South Africa is known to have one of the strongest economies in the region, it is essential to note that ours is the first study to endeavour to identify HPESUs in SSA generating insights that can inform future initiatives.

Part of the work of this research is to begin conversations across SSA’s HPESUs so that the experiences from within the SSA context can be shared regionally and internationally. Hopefully this initial work will help directors of HPESUs across the region connect with each other and engage in this information exchange, catalyzing a snowball of networking connections so that other HPESUs in SSA can be reached, leading to greater interconnectedness and fostering increased mutual support. If we acknowledge that the growing number of HPESUs in SSA has the potential to foster scholarship in the field [3], particularly work that can respond to the region’s unique context, and specific healthcare challenges [26], then providing the granular analysis of their units such as is offered in this study is important. It can enable HPESU leaders to better understand and serve their units, including leveraging the sort of resources and funding that they need to provide sustainability and foster future growth.

## References

[1] Davis MH, Karunathilake I, Harden RM (2005). AMEE education guide no. 28: the development and role of departments of medical education. Med Teach.

[2] Gruppen L (2008). Creating and sustaining centres for medical education research and development. Med Educ.

[3] Kiguli-Malwadde E, Talib ZM, Wohltjen H (2015). Medical education departments: a study of four medical schools in Sub-Saharan Africa. BMC Med Educ.

[4] Varpio L, Bidlake E, Humphrey-Murto S, Sutherland S, Hamstra SJ (2014). Key considerations for the success of medical education research and innovation units in Canada: unit director perceptions. Adv Health Sci Educ.

[5] Varpio L, O’Brien B, Hu W (2017). Exploring the institutional logics of health professions education scholarship units. Med Educ.

[6] O’Brien BC, Irby DM, Durning SJ (2019). Boyer and beyond: an interview study of health professions education scholarship units in the United States and a synthetic framework for scholarship at the unit level. Acad Med.

[7] Varpio L, Gruppen L, Hu W (2017). Working definitions of the roles and an organizational structure in health professions education scholarship: initiating an international conversation. Acad Med.

[8] Agyepong IA, Sewankambo N, Binagwaho A (2017). The path to longer and healthier lives for all Africans by 2030: the Lancet Commission on the future of health in sub-Saharan Africa. Lancet.

[9] Mullan F, Frehywot S, Omaswa F (2012). The Medical Education Partnership Initiative: PEPFAR’s effort to boost health worker education to strengthen health systems. Health Aff.

[10] Chen C, Buch E, Wassermann T (2012). A survey of Sub-Saharan African medical schools. Hum Resour Health.

[11] Murray CJ, Lopez AD (1997). Alternative projections of mortality and disability by cause 1990–2020: Global Burden Of Disease Study. Lancet.

[12] Kassebaum NJ, Bertozzi-Villa A, Coggeshall MS (2014). Global, regional, and national levels and causes of maternal mortality during 1990–2013: a systematic analysis for the Global Burden of Disease Study 2013. Lancet.

[13] Murray CJ, Vos T, Lozano R (2013). Disability-adjusted life years (DALYs) for 291 diseases and injuries in 21 regions, 1990–2010: a systematic analysis for the Global Burden of Disease Study 2010. Lancet.

[14] Braun V, Clarke V (2006). Using thematic analysis in psychology. Qual Res Psychol.

[15] Kiger ME, Varpio L (2020). Thematic analysis of qualitative data: AMEE guide no. 131. Med Teach.

[16] Braun V, Clarke V (2013). Successful qualitative research: a practical guide for beginners.

[17] United Nations, Statistics Division. Standard country or area codes for statistical use.. https://unstats.un.org/unsd/methodology/m49/. Access date: 5 May 2020.

[18] Monekosso G (2014). A brief history of medical education in Sub-Saharan Africa. Acad Med.

[19] Mullan F, Frehywot S, Omaswa F (2011). Medical schools in sub-Saharan Africa. Lancet.

[20] Malterud K, Siersma VD, Guassora AD (2016). Sample size in qualitative interview studies: guided by information power. Qual Health Res.

[21] Varpio L, Paradis E, Uijtdehaage S, Young M (2020). The distinctions between theory, theoretical framework, and conceptual framework. Acad Med.

[22] DiMaggio PJ, Powell WW (1983). The iron cage revisited: institutional isomorphism and collective rationality in organizational fields. Am Sociol Rev.

[23] Boxenbaum E, Jonsson S (2017). Isomorphism, diffusion and decoupling: concept evolution and theoretical challenges. The Sage handbook of organizational institutionalism.

[24] Tekian A (2014). Doctoral programs in health professions education. Med Teach.

[25] Tekian A, Harris I (2012). Preparing health professions education leaders worldwide: a description of masters-level programs. Med Teach.

[26] Van Schalkwyk S (2017). Scholarship for Africa: are we taking it seriously enough?. Afr J Health Prof Educ.

